# Deciphering *Trichoderma*–Plant–Pathogen Interactions for Better Development of Biocontrol Applications

**DOI:** 10.3390/jof7010061

**Published:** 2021-01-18

**Authors:** Alsayed Alfiky, Laure Weisskopf

**Affiliations:** 1 Department of Biology, University of Fribourg, Rue Albert-Gockel 3, 1700 Fribourg, Switzerland; laure.weisskopf@unifr.ch; 2 Genetics Department, Faculty of Agriculture, Tanta University, Tanta 31511, Egypt

**Keywords:** fungal chemical ecology, *Trichoderma*, mutualistic, mycoparasitism, plant defense, ISR, secondary metabolite, volatile organic compounds (VOC)

## Abstract

Members of the fungal genus *Trichoderma* (Ascomycota, Hypocreales, Hypocreaceae) are ubiquitous and commonly encountered as soil inhabitants, plant symbionts, saprotrophs, and mycoparasites. Certain species have been used to control diverse plant diseases and mitigate negative growth conditions. The versatility of *Trichoderma*’s interactions mainly relies on their ability to engage in inter- and cross-kingdom interactions. Although *Trichoderma* is by far the most extensively studied fungal biocontrol agent (BCA), with a few species already having been commercialized as bio-pesticides or bio-fertilizers, their wide application has been hampered by an unpredictable efficacy under field conditions. Deciphering the dialogues within and across *Trichoderma* ecological interactions by identification of involved effectors and their underlying effect is of great value in order to be able to eventually harness *Trichoderma*’s full potential for plant growth promotion and protection. In this review, we focus on the nature of *Trichoderma* interactions with plants and pathogens. Better understanding how *Trichoderma* interacts with plants, other microorganisms, and the environment is essential for developing and deploying *Trichoderma*-based strategies that increase crop production and protection.

## 1. Introduction

Heavy reliance on synthetic pesticides and fertilizers to support crop production has negatively impacted the environment and the health of humans and ecosystems [1,2]. Judicious exploitation of beneficial plant-microbe partnerships that have been evolutionally fine-tuned can help sustainably meet steadily increasing global demand for plant products. Biocontrol of plant diseases represents the most widely deployed form of such applications. Certain bacteria (e.g., *Pseudomonas*, *Paenibacillus*, *Streptomyces*, *Bacillus*) and fungi (e.g., *Trichoderma*, *Glomus*) confer one or more of the following benefits to plants: (a) protection from diverse pathogens and pests both directly (suppression of their growth or parasitism) and indirectly (induction of plant defense); (b) increased abiotic stress tolerance; and (c) enhanced growth. *Trichoderma* is one of the most extensively studied biocontrol agents (BCAs). The versatile lifestyle and nutritional adaptability of *Trichoderma* spp. enable members of the genus to occupy a wide range of ecological niches. Several species have proven able to form partnerships with different host plants as beneficial avirulent symbionts or endophytes [3,4,5]. Symbiotic relationships between host plants and *Trichoderma* spp. provide both parties with benefits. *Trichoderma*-based products have been commercialized as biocontrol agents (BCAs) to control a wide assortment of crop pathogens and as bio-fertilizers or growth-enhancers to promote plant growth [6]. The host plant roots provide *Trichoderma* with a suitable habitat and food; in return, they gain positive modulation of growth, yield, and stress tolerance. In complex multi-species communities sharing the same ecological niche, several important inter- and cross-kingdom communications are expected to take place [7,8,9]. Enhanced understanding of the molecular mechanisms underlying *Trichoderma*–plant and –fungal interactions is fundamental to judiciously harness *Trichoderma* full potential to support plant growth and health. Here, we reviewed how *Trichoderma* spp. directly and indirectly affects plant growth and health. 

## 2. *Trichoderma*-Plant Interactions

As opportunistic, avirulent plant symbionts, *Trichoderma* spp. have developed a wide array of strategies to build a mutually beneficial relationship with plants. This form of cross-kingdom communication is achieved through chemical signaling, in which fungi produce chemical compounds that alter plant transcriptome, proteome, and metabolome [10,11]. The outcome of such interactions is often favorable for plants in the form of improved growth and elevated tolerance to biotic and abiotic stresses. In this context of interactions, *Trichoderma* secretes a plethora of effectors to modulate plant growth and immunity. Proteins, small RNAs and different classes of secondary metabolites (SM) including VOCs (volatile organic compounds) have been documented to play different critical roles in *Trichoderma*-plant interactions [12].

### 2.1. Trichoderma as Plant Growth Regulators

Having the ability to regulate plant growth and physiology is a key feature of several members of the genus *Trichoderma*. They exert such effect through their abundant secretome of which specific proteins and SMs were found to target specific plant components regulating different physiological processes (Table 1). *Trichoderma*-mediated plant growth regulation could either be the direct effect of released molecules on plants or, indirect effect to *Trichoderma*’s impact and modification of the surrounding environment, e.g., modifying soil microbiome or lowering soil pH making the macro- and micronutrients more available to plants [13]. Such ability of *Trichoderma* spp. to regulate key pathways and physiological processes in plants could prove to be vital, especially under stress conditions where *Trichoderma* spp. can help alleviate such pressure. *T. yunnanense* and *T. afroharzianum* were reported to have the ability to enhance net photosynthesis, water use efficiency. and increased wheat (*Triticum aestivum* L.) biomass under salt stress [14]. Similarly, *T. longibrachiatum* ameliorated the negative effects of salt on wheat growth through activation of the antioxidative defense systems [15]. Many recent reports have detailed the ability of *Trichoderma* spp. to modulate physiological, biochemical, and molecular mechanisms in a wide assortment of plants under various growth conditions, all of which have indicated the huge potential of members of this genus to be used as biostimulants, biofertilizers, and biocontrol agents in agriculture [16,17,18,19].

#### 2.1.1. Root Colonization by *Trichoderma* spp.

The uncovered molecular dialogue between host plants and *Trichoderma* during root colonization revealed interesting mechanisms of cross-kingdom communications. During the asymptomatic colonization of plant roots by *Trichoderma*, the host plant activates several physical or biochemical responses, which limit the invading fungus to a few root cortical cell layers in plant roots. These responses include reinforcing and modifying plant cell walls, as well as producing reactive oxygen species and antimicrobial SMs [20,21,22,23]. The accumulated evidence suggests that *Trichoderma* have evolved ingenious mechanisms to hijack the hormone-regulated plant immunity in order to establish a prolonged mutualistic association. *Trichoderma* achieves host tolerance by suppressing plant defenses through the action of various effectors [23]. *Trichoderma*-mediated global transcriptome and proteome changes in plants have been recorded and their effect on development, growth modulation, and defense were at the center of several recent articles [24,25,26,27]. Research in this area has provided several meaningful insights revealing sophisticated molecular mechanisms of interaction between *Trichoderma* and their host plants [28,29,30]. It was reported that *T. virens* reduced maize (*Zea mays* L.) secretome by 36% during root colonization including major families of glycosyl hydrolases and peroxidases participating in plant defense against microbial invasion. In return, *T. virens* secretes a plethora of proteins and SMs performing different functions to establish root colonization in a synergistic synchronized manner. Such functions include secretion of cell wall degrading enzymes to facilitate entry to plant roots, antioxidant proteins to counteract the plant defense-induced oxidative burst consisting of superoxide, hydroxyl radical and hydrogen peroxide and several SMs and effector-like proteins that interfere with the homeostasis of plant hormones playing important roles in defense [31]. In a similar manner, extended transcriptome changes in *Arabidopsis thaliana* associated with transient repression of the plant immune responses and downregulation of defense genes in the roots was reported during *T. asperelloides* root colonization [32]. In agreement with these findings, defense-related genes in aerial parts of *A. thaliana* under the control of the salicylates–salicylic acid (SA) and jasmonic acid (JA) signaling pathways appeared to be downregulated during the early stage of root colonization by *T. harzianum* [33]. Proteome changes in shoots of maize during root colonization by *T. harzianum* showed that although *T. harzianum* was only present in roots, numerous proteins (including those involved in biotic and abiotic responses) were induced in foliar tissues [34]. Comparison between *T. harzianum*-treated, *Ganoderma boninense*-treated, and untreated palm oil plants for transcriptome dynamics revealed that three 1-aminocyclopropane-1-carboxylate (ACC) oxidase genes were downregulated, while another was upregulated in *Trichoderma*-treated oil palm roots compared to those of untreated plants. Although the same enzymes were activated in *Ganoderma*-treated plants, a different set of genes was activated in *Trichoderma*-treated plants, which suggests that elicited plant genes are differentially regulated by different fungal signals. The genes encoding 12-oxo-phytodienoic reductase were downregulated in *Trichoderma*-treated oil palm roots, indicating the downregulation of JA biosynthesis. Moreover, 12-oxo-phytodienoic acid is a cyclopentenone precursor of JA required for the wounding response [35] and the downregulation of the enzymes might therefore account for the asymptomatic colonization of plant roots by *Trichoderma* [35]. Taken together, these findings suggest that *Trichoderma* induce broad-spectrum suppression of host plant innate immunity in an attempt to maneuver and evade the plant defense responses during the initial stages of the interaction to successfully colonize the roots and establish a symbiotic relationship with the Plant 

#### 2.1.2. Alteration of Plant Hormonal Homeostasis

**Figure 1:**
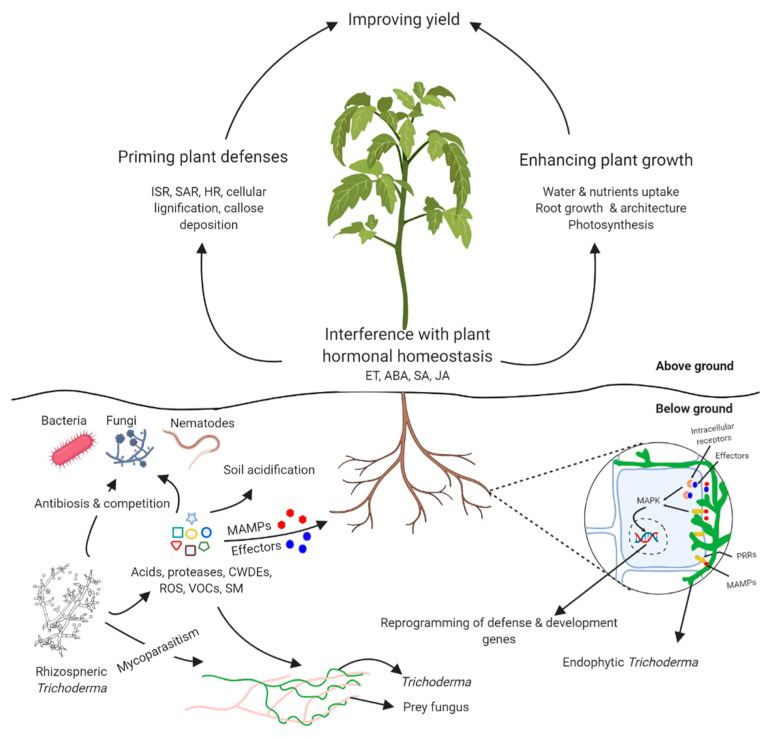
*Trichoderma*–plant–pathogen interactions map. *Trichoderma* produces recognition molecules, i.e., MAMPS (microbe-associated molecular patterns) and effectors, which bind to the PRRs (pattern recognition receptors) and intracellular receptors activating mitogen-activated protein kinase (MAPK) cascades, leading to the reprogramming of several plant pathways related to defense, i.e., induced systemic resistance (ISR), systemic acquired resistance (SAR), and hypersensitive response (HR), as well as interfering with hormonal homeostasis, i.e., ethylene (ET), abscisic acid (ABA), salicylic acid (SA), and jasmonic acid (JA) pathways. *Trichoderma*-pathogen interactions takes several forms through the action of its diverse secretome containing volatile organic compounds (VOC), cell wall degrading enzymes (CWDE), reactive oxygen species (ROS), and antimicrobial secondary metabolites (SM). By forming these interactions, *Trichoderma* increases plant fitness and tolerance against biotic stress either by priming plant defenses, increasing plant growth, or releasing pathogen pressure leading to the enhanced growth response.

Plant hormones play pivotal roles in the regulation of the complex and interconnected immune signaling networks, providing a huge potential for rapid response and adaptation to various conditions [36]. Moreover, the finely tuned plant hormonal homeostasis required for proper development, reproduction, and response to various stress conditions can be influenced by microorganisms synthesizing phytohormones. Phytohormones include different categories such as auxins, cytokinins, ethylene (Et), gibberellins, and abscissic acid (ABA), as well as hormone-like substances such as brassinosteroid, oligosaccharines, bioamines, salicylates–salicylic acid (SA), and jasmonic acid (JA) [37]. Microbially-derived phytohormones can help plants withstand biotic and abiotic stress conditions. The roles of exogenous phytohormone supplementation through plant-microbe interaction has been the focus of numerous studies [38,39,40,41]. Perception of *Trichoderma*’s secretome by plant roots is a first step in chain reactions that directly interfere with plant growth and physiology (Figure 1). Several members of *Trichoderma* spp. can produce phytohormones (auxin, gibberellin) and the enzyme 1-aminocyclopropane-1-carboxylic acid (ACC) deaminase can regulate the level of plant ethylene [37]. Moreover, volatiles from *T. asperellum* IsmT5 upregulated the plant SA production by 61% and ABA by 40% compared to plants not exposed to volatiles [42]. Isolates of different *Trichoderma* spp. which promote plant growth was shown to influence the phytohormone signature in melon plants, which led to increased auxin and decreased cytokinin and ABA contents. By evaluating the relationship between observed plant phenotype and hormonal variables, principal component analysis suggested strong association between auxin induction and *Trichoderma*-induced plant growth promotion [43]. These results suggest that alteration of plant hormonal homeostasis is a key mechanism by which *Trichoderma* can interfere with plant physiology and improve plant fitness. 

#### 2.1.3. *Trichoderma*-Produced VOCs as Plant Growth Regulators

In addition to direct root colonization and the emission of non-volatile compounds, the release of VOCs produced by *Trichoderma* can also alter plant growth and development. Several studies on the effects of VOCs from different *Trichoderma* spp. or strains on plant growth and physiology revealed that the effect is likely strain/species-specific. Volatiles from certain *Trichoderma* spp. and strains improved *A. thaliana* growth, plant biomass, chlorophyll content, and lateral root formation, while VOCs emitted by other species and strains had a negative effect [44]. Similar findings were reported for VOCs from *T. asperellum* T1 on lettuce plants as positive correlation between T1 VOCs and plant growth traits was reported [45]. Similarly, in another study, it was reported that *Trichoderma* VOCs were found responsible for inducing root branching and root hair formation in a manner similar to the changes observed in plants exposed to low iron availability [46]. Analysis confirmed that *Trichoderma*’s volatile compound 6-pentyl-2H-pyran-2-one (6PP) promoted plant growth and regulated root architecture in a specific and dose-dependent manner [47]. Through genetic screening of wild type seedlings, as well as auxin- and ethylene-related mutants, *A. thaliana* genes controlling root architectural responses to 6PP were identified and the results suggest that root responses to 6-PP involve components of auxin transport and signaling, and the ethylene-response modulator EIN2.

**Table 1:** Example of molecules secreted by *Trichoderma* spp. that modulate plant growth, defense, and physiology.

Molecule	Producer	Tested Plant	Effect and Known or Suggested Mode of Action	Reference Number
Volatile				
6-pentyl-α-pyrone (6PP)	*T. asperellum*	Arabidopsis	Pre-exposure activated defense responses and reduced symptoms against *Botrytis cinerea* and *Alternaria brassicicola*	[42]
6PP	*T. harzianum* *T. atroviride*	Tomato	Increased plant height, leaf area, developed roots system, and lycopene content in fruits	[48,49]
1-octen-3-ol	*T. virens*	Arabidopsis	Enhanced plant resistance against pathogens by activating JA/ET-dependent defense pathways	[50,51]
Trichodiene	*T. harzianum*	Tomato	Upregulated defense related genes in plants especially the SA-related genes.	[52,53]
Non-volatile				
Xylanase Xyn2/Eix	*T. viride*	Tobacco, Tomato	Elicited ethylene biosynthesis and hypersensitive responses	[54]
Hyd1 hydrophobin	*T. harzianum*	Maize	Elicited plant defense responses	[55]
Cellulases	*T. harzianum*	Maize	Induced ISR in plants via ET or JA pathways	[56]
Isoharzianic acid (iso-HA)	*T. harzianum*	Tomato	Improved seed germination and induced disease resistance.	[57]
Harzianolide	*T. harzianum*	Tomato	Promoted seedling growth and induced expression of defense related genes.	[58]
hydrophobin-like elicitor (SM1)	*T. virens*	Maize Cotton	Induced defense against plant pathogens.	[59,60]
Epl1	*T. asperellum* *T. harzianum*	PdPap, Tomato	Elicited plant defense Altered *B. cinerea* virulence	[61,62]
Swollenin	*T. asperellum*	Cucumber	Enhanced local defense responses and plant root colonization by *Trichoderma*.	[63]
Hydrophobin	*T. longibrachiatum*	Tobacco, Tomato	Elicited ISR and activated defense-related responses involving ROS and other compounds and stimulated root formation and growth.	[64]
Harzianic acid	*T. harzianum*	Tomato	Enhanced plant growth and increased seed germination rate	[57,58,59,60,61,62,63,64,65]
Cremenolide	*T. cremeum*	Tomato	Promoted plant growth	[66]

### 2.2. Induction of Plant Defenses by Trichoderma

Having to endure several biotic stresses from invading pathogens such as bacteria, fungi, or insects, which negatively impact growth and disrupt vital physiological processes, plants have evolved multiple mechanisms to counteract the prejudicial effect associated with such stress. Given that biological agents are far from being equal in terms of their modes of action and impact on plant health, plants have developed several scenarios to appropriately respond to microbial invasion. During some scenarios, plant defense responses are limited to the local infection areas, while other scenarios include upgrading the defense readiness of the entire Plant Within this second type of scenario, plants upgrade their defenses in tissues not affected by the initial attack to ward off secondary waves of attack, conferring a fitness advantage. In particular, two of the most extensively studied mechanisms for plant induced defenses are pathogen-induced systemic acquired resistance (SAR) and induced systemic resistance (ISR) triggered by mutualistic microbes such as *Trichoderma* spp. [60,67]. A significant body of recent literature has documented the potentiation and stimulation of plant defense responses by *Trichoderma* spp. [68,69,70]. In addition to their ability to directly antagonize plant pathogens and promote plant growth, several *Trichoderma* spp. can interfere with signaling networks in their host plants to improve disease resistance and stress tolerance. Chili pepper (*Capsicum annum* L.) treated with *T. harzianum* (as foliar spray), *T. asperellum* (as seed treatment) or combined application displayed enhanced defense and immunity against the fungal pathogen *Colletotrichum truncatum* through reprogramming of plant defense network in a strain- and application-dependent manner [27]. In pathogen presence, either single or dual consortia-treated chili plants showed varying augmentation of phenolic compounds such as caumeric acid, capsaicin, myrcetin, salicylic acid, and quercetin, which correlated with enhanced host plant immunity. The two *Trichoderma* spp. applied through different methods differentially affected expression of plant defense genes PR1 and PR2, indicating that both strains have different ways of eliciting plant immune response. 

To reveal the mechanisms by which *T. hamatum* induces tomato (*Solanum lycopersicum* L.) resistance to the bacterial pathogen *Xanthomonas euvesicatoria,* high density oligonucleotide microarray analysis revealed 41 differentially expressed genes associated with stress, cell wall modification, and signaling as well as RNA, DNA, and protein metabolism [71]. *T. harzianum* Epl-1, a protein elicitor of the cerato-platanin protein family, elicited the expression of genes involved in the SA defense pathway in tomato plants in the presence or absence of the pathogen. This elicitor also downregulated the expression of virulence genes of *Botrytis cinerea* especially those involved in the biosynthesis of botrydial, an important phytotoxic sesquiterpene metabolite, suggesting a key role for this protein in *T. harzianum* biocontrol interactions [62].

Pathogen-associated molecular patterns (PAMPs) or microbe-associated molecular patterns (MAMPs) refer to pathogenic or microbial effectors associated with plant health, development, and physiology [72]. ISR activation is a key outcome in plants triggered with MAMPs [73]. Plant recognition of microbial effectors by pattern-recognition receptors (PRRs) is considered the first step towards an effective immune response [74]. These PRRs play important roles in the perception of evolutionarily conserved (e.g., flagellin and chitin) or species-specific (e.g., ethylene-inducing-xylanase from *T. viride*) microbial effectors, wherein they transmit signals to trigger the PAMP/MAMP immune response [72,74].

*T. harzianum* treatment reduced disease severity and enhanced immunity and grapevine defense against *Plasmopara viticola*, the causal agent of grapevine downy mildew without directly inhibiting *P. viticola* sporangia germination, in a manner similar to the effect of SAR inducer benzothiadiazole (BTH) [75]. Comprehensive proteome analysis for *T. harzianum* induced resistance in grapevine revealed that most affected proteins belonged to signal transduction pathways suggesting MAMP-triggered immunity accompanied with rapid production of ROS and callose at infection sites [76]. Several studies aimed to dissect the molecular dialogue underlying the plant immune response to *Trichoderma*’s effectors. In this regard, it was confirmed that *Trichoderma* spp. are potent producers of lytic enzymes such as cellulases breaking down plant cell walls. *Trichoderma* cellulases have also been reported to induce ISR in plants via ET or JA pathways [77,78]. Consistently, the cellulase-like proteins *Thph1* and *Thph2* from *T. harzianum* triggered ISR in maize leaves while fungal ΔT*hph1*- or Δ*Thph2*-deletion mutant failed to activate maize immune response against an attack by the pathogenic fungus *Curvularia lunata* [56]. Moreover, the transcription factor MYB72 was upregulated in *A. thaliana* roots upon colonization by *T. asperellum*, while *A. thaliana myb72* mutants failed to express *Trichoderma*-triggered ISR against different shoot pathogens, indicating an essential role for MYB72 in the onset of *Trichoderma*-induced ISR in plants [79]. Elicitation of systemic immunity against downy mildew and modulation of pearl millet physiology in response to association with *T. hamatum* was also reported [80]. Cell wall lignification and callose deposition in addition to enhanced activities of several defense enzymes such as glucanase, peroxidase, phenylalanine ammonia-lyase, and polyphenol oxidase were observed in *T. hamatum*- treated plants in comparison to the untreated control. The increased activities of several SA-inducible genes such as glucanase, peroxidase, polyphenol oxidase, phenylalanine ammonia-lyase, and hydroxyproline-rich glycoproteins suggest a possible link between accmulation of SA in *Trichoderma*- treated plants and systemic resistance.

*Trichoderma*-mediated induction of plant defenses provides protection against invading microbial pathogens of diverse classes as well as insects and nematodes. Pre-treatment of *A. thaliana* seedlings with *Trichoderma* VOCs induced protection against *B. cinerea* and reduced disease severity and pathogen proliferation in the leaves three weeks after transplanting. Interestingly, micrografting of plant roots exposed to *Trichoderma* VOCs on a non-exposed scion revealed that the perception of the volatile signals by the roots was necessary to prime plant shoot defense mechanisms in a systemic manner [46]. Strains of *T. viride* and *T. harzianum* with biocontrol potential to protect rice plants (*Oryza sativa* L.) against *Rhizoctonia solani* induced detrimental morphological and physiological changes in the pathogen hyphae, including swelling, knotting, crumpling, flattening, shriveling, bursting, and cytoplasm leaking. Furthermore, accumulation of defense-related biomolecules in plants such as phenylalanine ammonia lyase (PAL), peroxidase, β-1,3-gluconase, chitinase, total phenolics content, polyphenol oxidase, and superoxide dismutase was also reported [81].

In addition to the response of plants to pathogens, *Trichoderma* can also induce plant defense against insect attack by the activation of plant defense-related pathways [82,83]. *T. harzianum* induced metabolic changes in tomato plants that indirectly counteracted the attack by the aphid *Macrosiphum euphorbiae* by attracting the parasitoid *Aphidius ervi* [84]. The concomitant presence of *T. harzianum* on plant roots and of aphids on the leaves induced parasitoid attraction towards the infested plants and the plants were shown to produce a different VOC profile in the presence of both *Trichoderma* and aphids than when either of them were alone, which may account for the observed changes of parasitoid flight behavior. Similar findings were obtained with *T. longibrachiatum*, which modified the VOCs released by tomato plants resulting in increased attractiveness to the natural enemies of the aphid *M. euphorbiae* [85].

## 3. Direct Interactions with Plant Pathogens

Microbial communities in the rhizosphere, including beneficial microbes and detrimental pathogens, are constantly competing for finite resources, such as limited space in soil or plant roots, as well as soil nutrients. Having an augmented arsenal of cell wall degrading enzymes (proteases, chitinases, lipases, glucanases, etc.), antibiotic SMs, powerful secretory, and cellular detoxification systems, in addition to their high adaptability to different environments, being heavily sporulating and fast growing are all factors contributing to establish and confirm the position of several *Trichoderma* spp. as “soil competent BCAs” against a wide spectrum of plant pathogens [86,87,88,89]. The powerful biocontrol machinery of *Trichoderma* spp. has been the main drive for exploring the efficacy of different species/isolates as potential BCAs, either as prophylactic or curative treatments against plant pathogens, especially when chemical fungicide treatments have proven to be ineffective or economically unfeasible. Different *T. harzianum* isolates were reported to successfully shield soybean (*Glycine max* L.) against charcoal rot disease caused by the soilborne pathogen *Macrophomina phaseolina* [90]. Tolerance to chemical fungicide is a prerequisite for *Trichoderma* spp. to join integrated pest management programs; therefore, evaluation of *Trichoderma* spp. resistance/tolerance to chemical fungicides and using various mutagenesis techniques to produce mutants with elevated fungicide-resistance was the center of numerous studies with promising results [91,92,93]. Through different mechanisms such as competition, antibiosis, and mycoparasitism, *Trichoderma* can directly antagonize plant pathogens relieving associated plants from their biotic stress and providing healthier growth conditions [94,95]. Mycoparasitism is a form of biotrophic interaction in which fungal parasites stay alive on the expense of other fungal hosts [96]. Successful commercialization of several *Trichoderma* BCAs relied heavily on their mycoparasitic ability to detect, antagonize, or kill other fungi. The process of mycoparasitism appears to be very complex, shared by several *Trichoderma* spp., but still involves host specific steps of first, detection of host fungi and later, unleashing a set of hydrolytic enzymes and mycotoxic secondary metabolites. Several genomic studies focusing on investigating the nature and mechanisms of mycoparasitism revealed that some species have larger arsenals of genes encoding hydrolytic enzymes and antifungal compounds [96,97], which might at least partially explain the variable biocontrol performance of different *Trichoderma* spp.

### 3.1. Early Perception of Host-Derived Signals Is Critical for Successful Mycoparasitism

At the molecular level, a crucial factor for successful mycoparasitism is the early detection of host signals. The molecular mechanisms underlying the activation of the biocontrol machinery in *Trichoderma* have been analyzed, and the currently accepted model suggests that lytic enzymes and antimicrobial secondary metabolites produced by *Trichoderma* in combination with the formation of infection structures are triggered by the presence of *Trichoderma*’s foes [98,99,100]. Such activation can only take place after *Trichoderma* either perceives host-derived external signals (e.g., components of the host cell wall, secreted metabolites) or experiences physical interaction with ligands located on the host surface [99]. Both *T. atroviride* and *T. virens* responded to *Rhizoctonia solani* already before any physical contact by activating mycoparasitism-related genes, which supports the hypothesis that diffusible host-released substances trigger the BCA’s antagonistic response [96]. Volatile organic compounds (VOCs), which are low molecular weight compounds comprising aliphatic and aromatic hydrocarbons, ketones, alcohols, esters, aldehydes, terpenes and other classes [101,102], might also be involved in such long-distance recognition. Indeed, VOCs emitted by other microorganisms were shown to affect the secretion of antibacterial and antifungal molecules in *Trichoderma* [103,104].

### 3.2. Mycoparasitism in Trichoderma Is a Conserved Mechanism with a Host-Dependent Processes

Transcriptome analysis revealed that different *Trichoderma* spp. upregulate both a common and a distinct set of genes during their contact with fungal pathogens pointing to the existence of alternative strategies to attack their hosts [105,106]. Gene expression analysis in *T. harzianum* upon interaction with the four fungal pathogens *Fusarium oxysporum, Colletotrichum capsici*, *Colletotrichum truncatum,* and *Gloesercospora sorghi* revealed significantly different responses to different hosts [107]. Using a computational approach, an algorithm was designed to predict potential effector proteins from *T. virens, T. atroviride,* and *T. reesei* [108]. One of the identified genes, *tacfem1,* an encoded protein with a CFEM (common in fungal extracellular membranes) domain, was upregulated during interaction with *R. solani* AG5 but not in the presence of *R. solani* AG2, revealing a host-specific response pattern. Heterotrimeric G proteins are important intracellular signaling proteins that participate in the perception of host signals as they activate the mycoparasitic attack in *Trichoderma* spp. [95,109,110,111]. They are activated by canonical G-protein coupled receptors (GPCRs) upon ligand binding [112,113]. It was shown that *T. atroviride* mutants missing *tga3*, a G-protein α subunit-encoding gene, were avirulent in direct confrontation assays with *R. solani* or *B. cinerea,* as they failed to secrete mycoparasitism-relevant hydrolytic enzymes and were unable to attach to host hyphae and form mycoparasitism-related infection structures in response to the host, suggesting a general role for Tga3 in host recognition [109]. Another gene with a possible role in the recognition of host-derived signals by *T. atroviride* is *gpr1*, which encodes a seven-transmembrane domain receptor belonging to the cAMP receptor-like class of fungal GPCRs [100]. Silenced *gpr1* transformants were avirulent in confrontation assays. Similar to ∆*tga3* mutants, they failed to attach to and attack their host fungi and were unable to induce the expression of the cell wall degrading enzymes upon contact with *R. solani*. Interestingly, the addition of exogenous cAMP to the confrontation plates was enough to restore the abilities for attachment and coiling around host hyphae in ∆*tga3* and *gpr1*-silenced strains. These findings indicate a role for *gpr1* in detecting host-derived signals and in activating intracellular regulatory targets, which results in activation of mycoparasitism-related features in *T. atroviride* via the cAMP pathway.

### 3.3. Trichoderma Proteins with Important Roles in Biocontrol

The lifestyle and biological functions of filamentous fungi are dictated by their ability to secrete extracellular effectors highlighting the importance of proteomic and metabolomic analysis to identify determinants playing key roles in *Trichoderma* spp. interactions and biocontrol efficacy. Several approaches for proteome analysis have been used to investigate and compare proteins produced by different members of *Trichoderma* spp. under a wide range of growth conditions [31,114,115,116,117]. A novel aspartic protease was identified and found to be differently upregulated in *T. harzianum* cultures amended with different fungal cell walls, suggesting a role in mycoparasitism and a proteomic response tuned to the fungal partner [118]. Proteome analysis of *T. harzianum* grown in the presence or absence of *Fusarium solani* or *B. cinerea* cell walls showed elevated protein content in pathogen-cell wall amended media with upregulated proteins belonging to mycoparasitism-related functions, such as chitinases, α and β 1,3-glucanases, glucoamylases, and proteases, suggesting that pathogen cell wall is indeed a main target of *T. harzianum* in the biocontrol mechanism [119,120].

### 3.4. The Roles of VOCs Released from Trichoderma in Microbial Interactions.

In addition to proteins, *Trichoderma* spp. produce a variety of secondary metabolites, including VOCs, which serve as info semiochemicals for protection and communication [48]. The use of antimicrobial VOCs from fungal biocontrol agents to inhibit microbial growth (mycofumigation) is a promising alternative to chemical fumigation. A significant body of recent literature aimed to evaluate the potential of different *Trichoderma* strains as producers of antimicrobial VOCs. Headspace analysis for VOCs from different members of *Trichoderma* spp. revealed inter- and intra-species variations for the ability to produce 6-n-pentyl-2H-pyran-2-one (6-PAP or 6PP) [121]. The volatile compound 6PP plays a key role in *Trichoderma*-plant interaction as it inhibits several phytopathogenic fungi, promotes plant growth, and alters root architecture inhibiting primary root growth and inducing lateral root development [48]. Additional volatile compounds identified from different *Trichoderma* spp. include hydrocarbons, alcohols, lactones, furanes, ketones, aldehydes, alkanes, alkenes, esters, aromatic compounds, heterocyclic compounds, and various terpenes [44,122,123,124]. Similarly, VOCs with antagonistic activities against several root-rot fungi were identified from *T. gamsii* [124].

### 3.5. Trichoderma Non-Volatile Metabolites and Their Role in Biocontrol

The ability of various members of *Trichoderma* spp. to produce secondary metabolites with antibiotic activity has attracted the attention of many researchers for a long time and, in addition to the VOCs mentioned above, various classes of SMs have been identified (e.g., non-ribosomal peptides such as peptaibiotics, siderophores, diketopiperazines-like gliotoxin/gliovirin, polyketides, terpenes, and isocyane metabolites) [88,125,126,127]. For more details regarding different SMs produced by different *Trichoderma* spp., we refer the reader to a recent review by Li et al., 2019 [128]. Although the mechanisms of interaction with plants and microbes are not fully elucidated yet for all identified SMs, it is believed that these compounds play important roles during biocontrol events due to their antimicrobial activities, which allows them to serve in chemical warfare against competitors and host fungi, while at the same time exhibiting plant growth-promoting and plant resistance-inducing activities [129,130]. Harzianic acid and harzianolide produced by *T. harzianum* were found to promote plant growth and harzianic acid, a tetramic acid with siderophore-like activity, which also inhibited diverse phytopathogens, including *Pythium irregulare, Sclerotinia sclerotiorum*, and *R. solani* [57,58,65].

The non-ribosomal synthesized peptaibols are a class of SMs that is specific to the mycoparasitic lineages of Hypocreales. These fungal oligopeptides (7–20 amino acids) are characterized by the presence of the nonproteinogenic amino acid alpha-aminoisobutyric acid (AIB) and generally exhibit antimicrobial activity against fungi and gram-positive bacteria [131,132]. Their antibiotic activity is based on membrane insertion of the peptaibol, which has a helical structure and forms ion channels and pores in the lipid bilayer [133]. Peptaibols were further shown to act synergistically with cell wall degrading enzymes [134], which results in a combined enzymatic lysis of the host cell wall and membrane leakage, processes that significantly contribute to the antagonistic action of *Trichoderma* mycoparasites [130].

## 4. Three-Partite Interactions: Crosstalk between Different Partners

**Figure 2:**
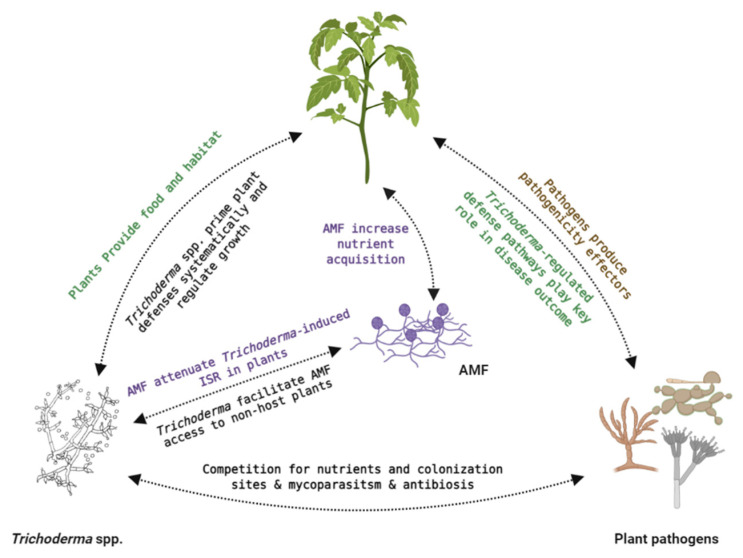
A schematic diagram displaying simplified glimpse of possible crosstalks between *Trichoderma* spp., plant pathogens, other beneficial microbes (i.e., arbuscular mycorrhizal fungi (AMF)) and plants.

Under field conditions, we cannot anticipate separate dual interactions between plant and pathogens, plant and BCA, or pathogen and BCA; rather, a complex network of interactions that involves multiple players is expected, and hence, investigating and understanding the three-way interactions between plant, BCA and pathogen is of high interest (Figure 2). Studying such tripartite systems can reveal important crosstalk between different interacting partners, which might not happen in bipartite interactions.

In plants, nitric oxide (NO) is important for several cellular functions including defense against microbes. Using a tripartite interaction system, it was revealed that *T. asperelloides* had the ability to switch off NO production in *A. thaliana* roots infected with *Fusarium oxysporum*. NO levels in *A. thaliana* roots were monitored by diaminofluorescein fluorescence visualization and revealed that when *A. thaliana* roots were infected with *F. oxysporum* alone, NO levels remained highly detectable for at least 120 min, while roots treated with *T. asperelloides* alone showed less intense and transient NO formation for 10 min, and finally roots coinfected with both *F. oxysporum* and *T. asperelloides* at the same time showed NO expression pattern similar to the *T. asperelloides* treatment. The drastic reduction in NO levels in plant roots during co-inoculation of *T. asperelloides* and *F. oxysporum* might explain failure of the pathogen to initiate disease symptoms in challenged plants [135]. 

Moreover, a tripartite interaction experiment between *A. thaliana, T. asperelloides,* and the leaf pathogen *Pseudomonas syringae* DC3000 (*Pst*) revealed that *A. thaliana* roots treated with *T. asperelloides* led to a significant increase in expression of several *Pst* defense genes in the plant leaves, confirming systemic priming of plant defenses by *T. asperelloides* root treatment. Among notably altered genes, in addition to several marker genes for Et/JA pathways that were upregulated, lipid transfer protein LTP4 encoding a pathogenicity related protein was highly upregulated. On the other hand, WRKY40, a transcription factor contributing to plant susceptibility to bacterial invasion, was downregulated [136]. Furthermore, proteomic analysis revealed major changes in *T. atroviride* proteome during double (*T. atroviride* + plant) or triple (*T. atroviride* + plant + pathogen) interactions with bean plants and the phytopathogens *B. cinerea* or *R. solani* compared to the growth of *T. atroviride* alone [137]. Many proteins were produced in the tripartite but not in the bipartite setup, suggesting fine-tuned response of the antagonist to the pathogen presence during its interaction with the Plant In addition to cell wall degrading enzymes, other upregulated *T. atroviride* proteins included a homolog of a 40 kDa heat shock protein, suggesting stress adaptation; chitin synthase involved in the synthesis of fungal cell wall, possibly to recover the damage caused by pathogen and plant enzymes; several cyclophilin family members playing various cellular roles; a hydrophobin and ATP-binding cassette transporters performing various roles connected with survival, stress adaption, plant growth promotion, and defense [12,64,86]. Mycoparasitic interactions between *T. atroviride* and plant pathogens *Pythium ultimum* and *R. solani* were visualized in situ on cucumber seeds using *gfp*-tagged *T. atroviride*, and revealed that activation of biocontrol related genes and formation of special infection structures happened during seed germination [138]. Similarly, a tripartite interaction experiment between *T. virens*, *F. oxysporum,* and tomato was used to study the mechanism by which *T. virens* regulated induced systemic resistance (ISR) in plants [139]. While treatment of two-week old tomato seedlings with either *T. virens* spores cultured on barley grains (BGS) or with *T. virens* cell-free culture filtrate (CF) significantly lowered *Fusarium* wilt disease incidence in tomato, further examination of disease incidence and defense gene expression revealed BGS primed ISR via JA, whereas CF primed ISR via SA. In another tripartite interaction study between *T. atroviride*, tomato seedlings, and the phytopathogen *Phytophthora cinnamomi*, *T. atroviride* proved able to outgrow the oomycete in the competition for available space and nutrients from both the medium and root exudates, shielding the seedlings from root rot symptoms. In the same study, it was suggested that plant root exudates are likely to play a key role in biocontrol by inducing *T. atroviride* growth and sporulation assisting the BCA in the competition for space and nutrients [140].

Plant defense responses to the invasion of pathogenic nematodes in the presence of *T. harzianum* T-79 revealed that T-79 differentially primed SA and JA mediated responses depending on the infection stage. Initially, T-79 primed the SA pathway, mounting a fast fortification of plant immunity against invasion. Later, when nematodes suppressed JA-mediated plant root defenses to establish infection, T-79 empowered plant immunity by priming this pathway to counteract the defense-suppressive effects of the nematodes. Finally, when nematodes established their infection, T-79 activated plant SA-mediated defenses in a local and systemic manner [141].

In addition to studying disease progression and mounted defense responses in plants, tripartite interaction experiments could be used to study microbial signaling not only between antagonists and pathogens but also between different beneficial microorganisms in the presence of plants. Along these lines, a study on the relationship between the two symbionts *T. harzianum* and the arbuscular mycorrhizal (AM) fungus *Glomus* sp. in potato plantlets revealed that the AM fungus increased phosphorus uptake; however, the host plant received less phosphorus as a result of the disruption of the external and internal AM fungal hyphal continuity due to *T. harzianum* mycoparasitism [142]. This highlights the importance of the nature of relationship and outcome of the interaction between different plant-beneficial microbes and how this complex network of interactions might directly or indirectly impact plant health and growth. *T. harzianum*’s ability to influence AM function and viability may also indirectly affect the plant performance by interfering with the source–sink relationship between the AM and its host Plant Similarly, it was revealed that co-inoculation of *T. harzianum* and AMF, resulted in attenuated priming of plant-induced systemic resistance compared with plants inoculated only with *T. harzianum* [143]. However, other reports indicated compatible relationship between *Trichoderma* spp. and AM fungi promoting plant growth and nutrient acquisition [144,145,146]. The combined application of AMF with *T. viride* improved P uptake and increased P content on *Helianthus annuus* roots and shoots in comparison to a single application of either of the beneficial microbes [146]. Combined application of both symbionts improved several growth parameters such as plant height, shoot biomass, root length, leaf area, and oil content. Similar findings were reported during combined inoculation of different AM and *T. viride* on onion (*Allium cepa*) [144]. Furthermore, *T. harzianum* T34 facilitated successful colonization of AM fungi to non-host plants such as *A. thaliana* and *Brassica napus* (rapeseed) and improved their productivity during combined applications [147]. Clearly further research is needed to explore the nature and molecular signaling between *Trichoderma* and AMF in regulating plant growth and defense pathways.

## 5. Conclusions and Perspectives

Despite coming a long way since *Trichoderma* was originally discovered in 1794 and its ability to be used as BCA was recognized in 1930s, extensive research and commercialization of some *Trichoderma* strains such as BCAs, the full potential of this remarkable genus has not yet been reached. To take the utilization of *Trichoderma* and other similar microbial BCAs to the next level, we must first try to better understand their physiology, behavior, and crosstalk with different pathogens and plants under different conditions. In vitro studies that search for, evaluate, and understand the antagonistic potential of different BCAs against various pathogens provided valuable information but another equally important field is the crosstalk of different partners sharing the same ecological niche. In this regard, we find that tripartite interaction studies between *Trichoderma*, plant and pathogens provide a valuable tool to better understand the underlying signaling during these interactions. However, we must note that even tripartite interaction studies should be considered as a reductionist approach, compared with real field conditions, which harbor more complex networks of interactions between different plants and their native microbiota, insects, BCAs, and pathogens. All of these interactions are also in turn affected by abiotic factors such as drought, temperature, humidity, chemical residues, and soil fertility. Recent advances in molecular biology such as genetic editing tools and advances in metabolomic analysis provide great opportunities for major breakthroughs to identify and validate important effectors playing key roles in the context of *Trichoderma* interactions. Advances in other areas such as formulations, shelf life, and compatibility with integrated pest management programs would increase the confidence and popularity of *Trichoderma*-based products among growers. Deciphering more of the biocontrol complex network of signaling under conditions that come as close as possible to natural conditions and identifying the spoken languages (proteins, VOCs, SMs, etc.) between *Trichoderma*, plants, and pathogens will enable us to successfully intervene with advanced molecular tools to improve the efficacy and consistency of *Trichoderma* biocontrol strategies and harness the full potential of this amazing genus.
